# Multifaceted Approaches in Epithelial Cell Adhesion Molecule-Mediated Circulating Tumor Cell Isolation

**DOI:** 10.3390/molecules30050976

**Published:** 2025-02-20

**Authors:** Dora Szerenyi, Gabor Jarvas, Andras Guttman

**Affiliations:** 1 Research Institute of Biomolecular and Chemical Engineering, Faculty of Engineering, University of Pannonia, 8200 Veszprem, Hungary; jarvas.gabor@mk.uni-pannon.hu; 2 CAPTEC Medical Ltd., 8200 Veszprem, Hungary; 3 Horváth Csaba Memorial Laboratory of Bioseparation Sciences, Research Center for Molecular Medicine, Faculty of Medicine, University of Debrecen, 4032 Debrecen, Hungary

**Keywords:** liquid biopsy, CTC, surface markers, EMT, immuno-affinity, label-free isolation

## Abstract

Circulating tumor cells (CTCs) are pivotal in cancer metastasis and serve as valuable biomarkers for diagnosis, prognosis, and treatment monitoring. Traditional CTC capture methods predominantly utilize the epithelial cell adhesion molecule (EpCAM) as a marker for isolation. However, the heterogeneity of these circulating cells and the epithelial-to-mesenchymal transition process (wherein epithelial cells acquire mesenchymal characteristics) limit the efficacy of EpCAM-based capture techniques. In this paper, we critically review the role of the EpCAM in CTC capture, explore the impact of epithelial-to-mesenchymal transition on EpCAM expression, and discuss alternative biomarkers and strategies to enhance CTC isolation. By evaluating the limitations of EpCAM-mediated capture and the challenges posed by epithelial-to-mesenchymal transition, we aim to provide insights into the development of more comprehensive liquid biopsy approaches for cancer management.

## 1. Introduction

Molecular recognition-mediated circulating tumor cell (CTC) capture is an advanced technique used to isolate and identify cancer cells circulating in the bloodstream. Since CTCs are shed from primary tumors and travel through blood vessels, they play a crucial role in the development of cancer metastasis, making them a valuable target for diagnostic purposes [1,2,3,4,5,6]. CTCs exhibit resistance to anoikis, a form of programmed cell death induced by detachment from the extracellular matrix. This resistance allows CTCs to survive in the bloodstream, facilitating their dissemination and metastatic potential [7,8]. In addition, CTCs can possess stem cell-like characteristics (including self-renewal and differentiation capabilities), related to epithelial-to-mesenchymal transition [9,10,11,12], when epithelial cells acquire mesenchymal traits [13]. Immune evasion by the expression of peptides that bind to class I molecules of the major histocompatibility complex [14], aggregation with platelets, and the formation of clusters also promote their survival and facilitate the metastatic process [15]. CTCs are typically larger than most cellular blood components [16], and often exhibit morphological heterogeneity compared to the more uniform morphology of normal blood cells [11,17]. CTCs exhibit distinct electrophysiological traits, which can be exploited for their isolation and characterization [18,19]. Cell stiffness is a biomechanical property that distinguishes, e.g., ovarian cancer cells from non-malignant cells [20,21]. Cancer cells exhibit higher mechanical resistance than their healthy counterparts and might be privileged to migrate into distant organs [20,22].

**Figure 1:**
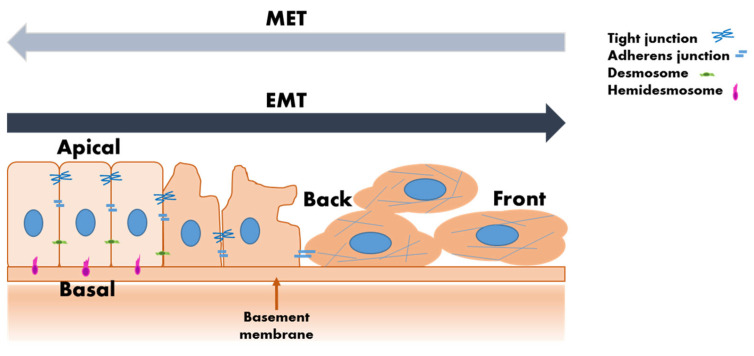
The epithelial-to-mesenchymal transition (EMT). The EMT is a cellular process throughout which epithelial cells acquire mesenchymal features, morphologies, and behaviors following the downregulation of epithelial phenotypes. The process is activated in response to signals that cells get from their microenvironment. EMT shows variability at the single-cell and population levels too. Instead of adapting mesenchymal markers and structures completely, cells often acquire a combination of epithelial and mesenchymal markers, morphologies, and behaviors. This process enhances the metastatic potential of malignous cells and makes their detection more difficult using EpCAM-dependent techniques.

Detecting and analyzing CTCs can provide critical insights into cancer progression and treatment efficacy (Table 1). The challenge lies in capturing these cells effectively from the blood, as they are present in extremely low concentrations among millions of normal blood cells [23,24,25,26,27]. The Epithelial Cell Adhesion Molecule (EpCAM) and other epithelial markers have been the cornerstone in the detection and capture of circulating tumor cells (CTCs) through liquid biopsy techniques [28,29,30,31]. However, their effectiveness is limited during epithelial-to-mesenchymal transition (EMT), a process where epithelial cells acquire mesenchymal traits, leading to the downregulation of epithelial markers [32,33]. EMT exhibits variability at both the individual cell and population levels. Instead of fully converting to a mesenchymal state, cells often acquire a mix of epithelial and mesenchymal markers, morphologies, and behaviors [13,34,35] (Figure 1). This transition enhances the metastatic potential of tumor cells but also hinders their detection using EpCAM-based methods [5]. Incorporating other markers into CTC detection or combining them with EpCAM is a reliable strategy to enhance the sensitivity and specificity of liquid biopsies [6,28], particularly in cases where EpCAM expression is diminished due to EMT. By targeting a combination of epithelial and mesenchymal markers, researchers can aim to develop more comprehensive and reliable methods for CTC isolation and analysis, thereby improving the prognostic and therapeutic management of cancer patients. CTCs with low or no EpCAM expression could serve as a valuable alternative for tumor analysis, even though they may be less prognostically significant compared to EpCAM high-expressing CTCs. They are especially useful when no CTCs can be detected using EpCAM-based methods [6].

The aim of this review is to reveal the role of the EpCAM in circulating tumor cell isolation, addressing its limitations due to the heterogeneity of CTCs and the epithelial-to-mesenchymal transition. The article explores alternative biomarkers and innovative strategies for CTC capture, evaluates EpCAM-based and independent enrichment techniques, and emphasizes the need for multifaceted approaches that combine analysis of molecular and biophysical characteristics to improve liquid biopsy sensitivity and specificity for cancer diagnostics, prognostics, and therapeutic monitoring.

## 2. EpCAM-Based CTC Enrichment

One of the most well-known antigens for epithelial-type CTCs is the epithelial cell adhesion molecule (EpCAM), a transmembrane glycoprotein, first reported in 1979 [36]. EpCAM plays a significant role in cell adhesion, signaling, migration, proliferation, and differentiation in epithelial cells [30,37,38,39,40]. Therefore, this protein has become a focal point in cancer research due to its high expression in many epithelial-derived cancers [31,41], where it is often overexpressed compared to normal tissues [39,40,42]. In healthy epithelial cells, the EpCAM mediates cell–cell adhesion and helps in maintaining the tissue structure and integrity. It plays a key role in the formation of tight junctions, which hold cells together within tissues. The EpCAM is also involved in regulating cell proliferation and differentiation through signaling pathways. While it is expressed at lower levels in most normal tissues, it becomes upregulated in regenerative or proliferative processes [30]. In many carcinomas, the EpCAM is overexpressed, a feature that has been linked to increased cell proliferation, migration, and invasion. This overexpression is a hallmark of various cancers, including breast, colon, prostate, ovarian, and pancreatic cancers [39]. In addition to its usual feature as cancer stem cell (CSCs) or CTC biomarker, the EpCAM is a relevant object of CTC capture and targeted therapy [4,42,43,44,45,46,47,48,49,50,51,52].

**Figure 2:**
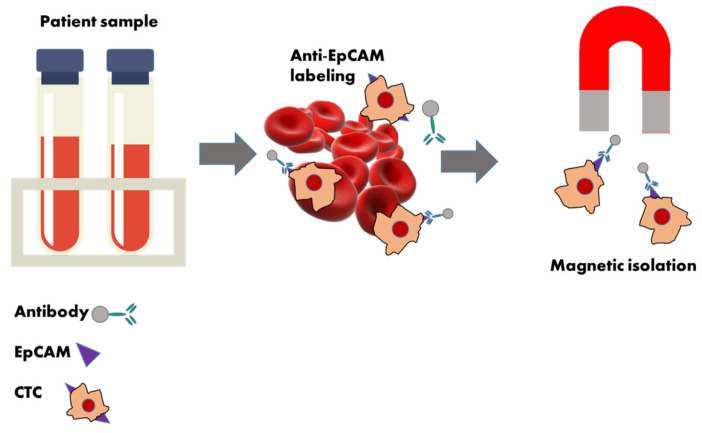
A schematic representation of the CellSearch method. This system is an FDA-approved liquid biopsy analysis device based on the positive selection of EpCAM^+^/CK^+^/CD45^−^/DAPI ^+^ (CS-CTC) CTCs. It operates by isolating CTCs from a 7.5 mL blood sample using immuno-magnetic partitioning. The technique is utilized for detecting and enumerating circulating tumor cells in patients with metastatic breast, prostate, and colorectal cancers.

Circulating tumor cells (CTCs) are promising biomarkers, as they can signify active systemic cancer. The current gold standard for CTC detection is the CellSearch (CS) system (Menarini Silicon Biosystems, Bologna, Italy), a liquid biopsy analysis device primarily based on positive selection, approved by the U.S. Food and Drug Administration (FDA) for detecting and enumerating circulating tumor cells in patients with metastatic cancers, including breast, prostate, and colorectal cancers [53,54,55,56,57] (Figure 2). It operates by isolating EpCAM^+^/CK^+^/CD45^−^/DAPI ^+^ (CS-CTC) CTCs from 7.5 mL blood samples using immunomagnetic separation [2,57,58,59]. Despite improvements, the sensitivity of the CellSearch system is still not always adequate, especially in cancers with low CTC counts. Detecting rare CTCs in early-stage cancers or in cases with minimal residual disease can be challenging [54,55]. The reliance on the EpCAM for CTC capture may lead to underestimation of CTCs undergoing epithelial–mesenchymal transition, during which EpCAM expression diminishes [60]. In addition, variability in sample handling and processing can also impact the reproducibility of the results. Standardizing protocols across laboratories is essential to ensure consistent and reliable outcomes. In summary, while the CellSearch system has significantly contributed to the field of liquid biopsy-based cancer recognition by enabling the detection and enumeration of CTCs, ongoing research aims to address its limitations and enhance its clinical applicability [53]. The IsoFlux system (Fluxion Biosciences, Inc., Alameda, CA, USA) is a semi-automated platform that enriches circulating tumor cells (CTCs) by combining EpCAM-based immunomagnetic positive selection with microfluidic technology, similar to the FDA-approved CellSearch system. Blood samples are mixed with antibody-coated magnetic beads, and then the prepared sample is introduced into the microfluidic cartridge, equipped with a cap that enhances cell recovery by providing a high-cell-density environment for the captured cells. As the sample flows through the cartridge, a magnetic field is applied, causing the magnetic beads bound to CTCs to adhere to the upper surface of the microfluidic channel. This magnetic capture ensures that CTCs are effectively separated from other blood components. After the capture phase, the cartridge is removed from the instrument, and the beads with the captured CTCs are retrieved from the cap. This mechanism ensures transfer efficiency, allowing for high-quality, viable cells to be collected for downstream analyses [61,62]. Studies have demonstrated an increase in recovery rates from 40% to 90% when using cancer cell lines with IsoFlux. This technology has been effectively utilized to isolate CTCs in patients with bladder, prostate, ovarian, liver, and pancreatic cancers [63,64]. CellCollector (Gilupi, Potsdam, Germany) is the first in vivo cell capture technique with CE approval, showing potential in detecting CTCs in cases of various cancer types, including lung, prostate, and neuroendocrine tumors. This device is a medical wire bearing anti-EpCAM antibodies directly placed in the bloodstream of patients through a catheter inserted in the vein of the arm. While it remains implanted in the vein for 30 min, it gets in contact with a larger volume of blood, allowing the capture of CTCs in vivo [54,65]. Earlier studies have shown that the CellCollector system may detect CTCs in a higher proportion of patients compared to CellSearch, which may be due to eliminating the problem of small sample volumes and reducing the need for extensive sample preparation [66]. For instance, in lung cancer patients, the CellCollector identified CTCs in 58% of cases, whereas CellSearch detected them in 27%. Similarly, in neuroendocrine tumor patients, the CellCollector found CTCs in 97% of cases, compared to 47% with CellSearch [66,67]. However, EpCAM dependence is still a concern, since the device potentially misses certain CTC populations. Also, the technique is much more invasive compared to CellSearch, because it requires the insertion of a permanent catheter into a vein, which may be less convenient for patients [65,68]. In summary, CellSearch is FDA-approved and widely used in clinical practice, while CellCollector is mainly utilized in research settings. Both technologies depend on EpCAM for CTC capture, which may limit their ability to detect CTCs undergoing EMT. This is a common limitation that researchers are currently addressing by exploring additional markers and methodologies.

### Integration of Other Biomarkers into EpCAM-Based CTC Enrichment

Other biomarkers used in specific types of cancers include human epidermal growth factor receptor 2 (HER2) and epidermal growth factor receptor (EGFR). HER2 (also known as ErbB2) is a member of the human epidermal growth factor receptor family, which is a group of tyrosine kinase receptors [45,69,70,71]. While HER2 does not have its own ligand-binding domain, it forms heterodimers with other receptors in the EGFR family [71,72]. This dimerization activates downstream signaling pathways involved in cell proliferation, survival, and differentiation [73]. HER2 is overexpressed in certain cancers, most notably breast cancer, as well as some cases of gastric, ovarian, and lung cancers [45,74,75]. Of all breast cancer cases, 10–20% are HER2 positive and the remaining 80–85% are considered HER2 negative, even though HER2 expression can be detected by immuno-histochemistry (IHC) [76]. HER2 overexpression is often associated with aggressive tumor behavior, poor prognosis, and increased risk of metastasis [75,77,78,79]. HER2 is used as a biomarker to capture and identify CTCs, and it is also a key target in cancer treatment [43,44,45,71,80,81]. Monoclonal antibodies, such as Trastuzumab and pertuzumab, are used to treat cancer by blocking HER2 signaling, leading to reduced tumor cell proliferation [70,75,78,79]. EGFR (also known as ErbB1 or HER1) is a transmembrane tyrosin kinase receptor [72,82]. Upon binding its ligand (e.g., epidermal growth factor), EGFR undergoes dimerization and autophosphorylation, which activates signaling pathways like the MAPK/ERK and PI3K/AKT pathways [82,83] driving cell division, survival, and migration [84]. It is often overexpressed or mutated in various cancers, such as lung [85], head and neck [86,87], colorectal [88,89,90], and glioblastoma [91]. Mutations lead to uncontrolled receptor activation and are associated with increased tumor growth and spread. EGFR is used to capture and label CTCs, especially in non-small cell lung cancer (NSCLC) and head and neck cancers [83,92,93,94]. Combining HER2 and EGFR with EpCAM in circulating tumor cell enrichment strategies enhances the capture of heterogeneous CTC populations, addressing the limitations of solely relying on EpCAM-based methods. By targeting multiple surface proteins, this approach captures CTCs that may not express EpCAM but do express HER2 or EGFR, thereby encompassing a broader range of tumor cell phenotypes [95]. Utilizing a combination of markers reduces false negatives associated with single-marker reliance and increases the likelihood of detecting CTCs with variable marker expression [28]. Isolating CTCs expressing specific markers like HER2 and EGFR enables detailed molecular characterization, aiding the selection of targeted therapies and monitoring treatment efficacy. Integrating antibodies against EpCAM, HER2, and EGFR in CTC isolation platforms improved capture rates and provided a more comprehensive profile of CTC populations in breast and non-small cell lung cancer patients [28,95,96]. However, the understanding of HER2 expression is evolving from a simple binary classification of HER2-positive and HER2-negative cancers to a wider range. This shift acknowledges that HER2 expression levels vary, influencing treatment decisions and outcomes; therefore, there is a need for novel biomarkers [97,98].

Mesenchymal markers could also be viable choices for detection and characterization of CTCs in liquid biopsy-based strategies. Activated leukocyte cell adhesion molecule (ALCAM) was identified as a significant marker for circulating tumor cells (CTCs) with low epithelial cell adhesion molecule (EpCAM) expression in pancreatic ductal adenocarcinoma as well as brain metastases of non-small cell lung cancer and validated as a novel alternative surface marker on EpCAM-low CTCs. Targeting N-cadherin, which is usually upregulated in EMT [99], has also been explored to improve the isolation of CTCs. Combining N-cadherin targeting with traditional epithelial markers like EpCAM has been shown to enhance CTC detection and broadened the variety of captured CTC phenotypes. For instance, a study demonstrated that combining EpCAM with N-cadherin-targeted isolation improved CTC detection and expanded the range of captured CTC phenotypes. However, caution is advised when targeting N-cadherin for CTC isolation, as it may also capture circulating endothelial cells (CECs), leading to false positives. To distinguish between CTCs and CECs, additional markers such as vascular endothelial–cadherin can be used [100]. Combining EpCAM with cell-surface vimentin (CSV) in circulating tumor cell enrichment could also be a promising strategy to enhance the capture of heterogeneous CTC populations, while addressing the limitations of relying solely on EpCAM-based methods [101,102,103,104,105]. Vimentin is an intermediate filament protein that is normally expressed in mesenchymal cells and is part of the cytoskeletal structure; it also provides structural integrity, supports cell shape, and is involved in cellular processes like migration and wound healing. Vimentin is a key player in EMT, a process where epithelial cells acquire mesenchymal characteristics. CTCs undergoing EMT are often more invasive and resistant to capture by traditional markers like EpCAM [106]. Anti-vimentin antibodies can be used to isolate these EMT-CTCs [107,108], which are crucial for studying the metastatic process [48,109,110]. The GenoCTC (Gencurix, Seoul, Korea) device utilizes microfluidic magnetophoresis and a specialized isolation chip with optimized ferromagnetic wire patterns to enrich circulating tumor cells using anti-human EpCAM beads, targeting mesenchymal–epithelial transition (MET) markers, or anti-human vimentin beads, targeting epithelial–mesenchymal transition (EMT) markers [111]. The On-chip Sort (On-Chip Biotechnologies, Tokyo, Japan) is an innovative benchtop cell sorter that utilizes disposable microfluidic chips for efficient cell sorting [112,113]. In this process, fluorescent antibodies are utilized for positive selection of different cells in the processed sample. Antibodies against a variety of markers, such as cytokeratin, vimentin, and CD45, can be used during the cell sorting. This method was successfully used in spike-in experiments comprising a series of lung cancer cell lines with different EpCAM expression levels [114]. Similar to the previous method, circulating tumor cells in peripheral blood from high-risk populations and cancer patients were enriched and identified using a positive sorting method that employed liposome magnetic beads targeting the epithelial cell adhesion molecule (EpCAM) and vimentin [101].

The M30 neoepitope is a specific fragment of cytokeratin 18 (CK18) exposed during early apoptosis, which serves as a valuable biomarker for detecting apoptotic epithelial cells, including circulating tumor cells (CTCs) [115,116,117,118]. A study demonstrated that M30-positive CTCs could be detected in over 70% of carcinoma patients, integrating the CellSearch system with M30-antibodies. The proportion of M30-positive CTCs varied between 50% and 80%. This finding suggests that monitoring M30 expression on CTCs can provide insights into disease progression and treatment response [116].

Integration of circulating tumor DNA (ctDNA) analysis in the test can improve the sensitivity and specificity of liquid biopsies, enabling more comprehensive tumor profiling. For instance, combining EpCAM-, or other surface-based CTC detection methods, with ctDNA analysis reportedly enhanced the diagnostic accuracy of, e.g., the sensitivity of primary lung cancer diagnosis, which may be clinically useful and could enhance early detection of the disease [119]. Combined use of CTC and circulating cell-free DNA (ccfDNA) in liquid biopsy tests has shown potential in metastatic breast cancer management. A study revealed that CTC and ccfDNA levels had a combined effect on patient outcomes. Patients with high levels of both CTC and ccfDNA exhibited increased mortality risk, compared to those with low levels of both CTC and ccfDNA [120].

## 3. EpCAM-Independent Circulating Tumor Cell Enrichment Strategies

### 3.1. Negative Selection

EpCAM-based positive selection methods directly bind to CTCs. However, they often co-isolate non-specific cells, leading to contamination by leukocytes or other blood cells. In addition, some CTCs may have low or no EpCAM expression due to the EMT process [13,121,122]. Negative selection does not rely on EpCAM expression, allowing for the capture of a broader range of CTC phenotypes using a technique that removes unwanted cells, rather than directly capturing the target cells [123,124]. This is typically achieved by using antibodies against markers found on non-target cells, such as CD45, a marker for leukocytes [125]. In the CellSearch^®^ system, the primary method for isolating CTCs is positive selection by using magnetic nanoparticles coated with antibodies against EpCAM. However, the system also employs CD45-specific antibodies, a marker expressed on leukocytes, and these are used to identify white blood cells during the analysis phase, rather than as a separate negative selection step. In other words, the CellSearch system does not incorporate a distinct negative selection phase. Instead, it depends on positive selection for CTC enrichment and uses CD45 staining to exclude leukocytes during the downstream analysis, thereby enhancing purity of the sample [126,127,128,129,130]. Negative selection eliminates CD45^+^ leukocytes, thereby reducing the non-specific background and allowing for a cleaner pool of CTCs for downstream analysis, enhancing the efficiency of EpCAM-dependent liquid biopsy methods. The use of superparamagnetic Dynabeads (Thermo Fisher Scientific, Waltham, MA, USA) that can be coated with antibodies to target specific cell surface markers (both anti-EpCAM for positive selection and anti-CD45 for negative selection), are a good example for this method [131]. When coated with anti-CD45 antibodies, Dynabeads can effectively bind to and remove CD45-positive leukocytes from a sample. This process enriches the remaining cell population for CTCs, thereby enriching viable and untouched target cells for detection and quantification studies. The RosetteSep (Stemcell Technologies™, Vancouver, BC, Canada) CTC Enrichment System also utilizes a negative selection approach to isolate circulating tumor cells from peripheral blood. This method involves crosslinking unwanted cells, such as leukocytes, to red blood cells (RBCs) to form rosettes whish are subsequently removed through density gradient centrifugation, resulting in an enriched population of CTCs. By not relying on positive selection markers like EpCAM, the RosetteSep technique preserves a broader spectrum of CTC phenotypes, including those with low or no EpCAM expression. A study published in 2022 highlighted the application of the RosetteSep system in enriching CTCs for downstream analyses. The researchers employed a bead-based CTC enrichment strategy, which facilitated the isolation of CTCs without the bias introduced by EpCAM-based selection methods. This negative selection technique is particularly advantageous for capturing CTCs that have undergone epithelial–mesenchymal transition, a process during which cells may downregulate EpCAM expression. By avoiding EpCAM-based positive selection, the RosetteSep system ensures a more comprehensive representation of CTC heterogeneity, which is crucial for accurate cancer diagnosis and monitoring [132,133,134].

Researchers developed a two-step process combining immunomagnetic bead-based negative selection with an ODEP-based (Optically Induced Dielectrophoresis) microfluidic device. ODEP is a technique that utilizes light patterns to create virtual electrodes on a photoconductive surface, enabling the manipulation of microscopic particles, including cells, without physical contact [135,136,137]. This approach first depletes CD45-positive leukocytes using magnetic beads, followed by the use of ODEP to further purify the CTCs. The method achieved high cell purity (81.6–86.1%) and maintained cell viability, facilitating downstream analyses [138,139]. A similar approach utilized negative selection to remove leukocytes, followed by ODEP in a microfluidic chip to isolate viable CTCs. This method significantly increased the purity of the resulting CTC fraction while maintaining their viability, making them suitable for further molecular and functional analyses [140].

The CTC-iChip, developed at the Massachusetts General Hospital, is an advanced microfluidic device designed to isolate circulating tumor cells from blood samples through a label-free, negative selection process [127,141]. The process begins with deterministic lateral displacement (DLD), a size-based separation technique that removes smaller components such as red blood cells and platelets from the blood sample. This step enriches the sample for nucleated cells, including CTCs and white blood cells (WBCs). Following DLD, the sample undergoes inertial focusing, aligning the remaining cells into a single stream within the microfluidic channel. This alignment is crucial for the precise application of downstream separation techniques. In the final step, the device employs magnetophoresis to deplete WBCs, which are labeled with magnetic particles via antibodies targeting specific markers (e.g., CD45, CD66b, and CD16). When subjected to a magnetic field, these labeled WBCs are deflected away from the main flow, leaving behind an enriched population of unlabeled, viable CTCs. By not relying on positive selection markers like EpCAM, the CTC-iChip captures a broader range of CTC phenotypes, including those that may have undergone epithelial–mesenchymal transition (EMT) and lack traditional epithelial markers. In addition, the negative selection approach preserves the viability of the isolated CTCs, making them suitable for various downstream applications, including molecular analyses and culturing. The device achieves significant depletion of non-target cells, resulting in high-purity CTC samples. The CTC-iChip can process blood samples at a rate of approximately 13 mL per hour, making it suitable for clinical applications where larger sample volumes are necessary. The ability to isolate viable CTCs without bias toward specific markers allows for comprehensive molecular characterization, aiding in personalized cancer treatment strategies. The efficiency and scalability of the CTC-iChip make it a promising tool for integrating liquid biopsy techniques into routine clinical practice [127,141,142,143,144,145,146].

Certain WBC subpopulations, particularly granulocytes, however, can express low levels of CD45 and may also be stained in a non-specific fashion for cytokeratin, leading to potential misclassification as CTCs. To alleviate this issue, researchers have explored the use of additional exclusion markers such as CD15, a marker strongly expressed on granulocytes, which has thus been identified as effective in distinguishing these cells from CTCs. Incorporating CD15 into the exclusion criteria, alongside a highly specific CD45 antibody, significantly reduces false positive rates. Flow cytometry analyses confirm the specificity of CD15 for granulocyte subpopulations, enhancing the accuracy of CTC detection. Implementing a dual-exclusion strategy that combines CD15 and CD45 antibodies, along with optimized cytokeratin antibody selection, has been shown to reduce false-positive rates from 25% to 0.2%. This approach underscores the importance of robust exclusion criteria and high antibody specificity in immunoassays for CTC identification. By effectively eliminating interfering WBC subpopulations, this method enhances the reliability of CTC detection, facilitating more accurate patient monitoring and potentially improving clinical outcomes [121]. Furthermore, the presence of CD45^+^/EpCAM^+^ cells also complicates CTC isolation and identification, hindering clinical translation. CTCs can form microemboli, comprising diverse phenotypic populations such as mesenchymal CTCs, and can aggregate into homotypic and heterotypic clusters. These clusters interact with other circulating cells, including immune cells and platelets, potentially enhancing the malignancy of the clusters. Notably, these microemboli represent a prognostically significant subset of CTCs [27].

### 3.2. Label-Free CTC Enrichment: A Challenger of Immune-Affinity-Based Methods

The Parsortix system (ANGLE plc, Guildford, UK) is a liquid biopsy technology for capturing and harvesting CTCs from blood samples, which sorts target cells based on their biophysical characteristics [33]. It features a microfluidic cassette that isolates cells according to their unique size and deformability, effectively differentiating them from other blood components. Unlike traditional methods that depend on specific surface markers, the Parsortix system does not require antibodies, allowing for the capture of a diverse range of CTC phenotypes, including those undergoing EMT [147,148,149]. This approach ensures that the viability of the captured CTCs is preserved, enabling a variety of downstream applications [150]. In May 2022, the Parsortix PC1 system achieved FDA clearance for isolating CTCs from blood samples of patients with metastatic breast cancer, marking a pivotal advancement in liquid biopsy technology and highlighting its promise for non-invasive cancer diagnostics and monitoring [147,149,151]. ClearCell FX1 is a similar automated system, developed by Biolidics Limited (Singapore). Utilizing a label-free approach, similar to the above, it preserves the integrity and viability of CTCs, facilitating various downstream analyses. The system employs Dean Flow Fractionation (DFF) within a microfluidic biochip (CTChip FR1), to separate CTCs based on size, deformability, and inertia, eliminating the need for antibody-based capture [152,153,154,155,156]. Label-free systems like Parsortix and ClearCell FX can capture a broader range of CTCs, including those with mesenchymal characteristics, whereas immunoaffinity methods may miss these due to marker variability. On the other hand, immunoaffinity methods often achieve higher purity by specifically targeting known markers, but they may not capture all CTC subtypes such as their label-free counterparts, which may co-isolate non-CTC cells, affecting purity [33]. All discussed CTC enrichment technologies are summarized in Table 2.

## 4. Conclusions

The isolation and analysis of circulating tumor cells (CTCs) are pivotal in advancing cancer diagnostics, prognostics, and therapeutic monitoring. Traditional EpCAM-based enrichment methods have significantly contributed to this field; however, their limitations, particularly concerning CTCs undergoing epithelial-to-mesenchymal transition (EMT), necessitate the exploration of alternative or complementary strategies. Incorporating additional markers such as HER2, EGFR, and vimentin into enrichment protocols has demonstrated enhanced capture efficiency and a more comprehensive representation of CTC heterogeneity. Furthermore, label-free technologies offer promising avenues by isolating CTCs based on biophysical properties, thereby circumventing the biases associated with marker-dependent methods. These methods generally preserve cell viability better, facilitating downstream analyses such as culture and molecular profiling. Immunoaffinity methods may impact cell viability due to antibody binding. In summary, the choice between label-free and immunoaffinity-based CTC isolation methods depends on the specific requirements of the study or clinical application, considering factors like the need for capturing heterogeneous CTC populations, desired purity levels, and further evaluation. The integration of negative selection techniques further refines CTC isolation by depleting non-target cells, thus enriching the sample for viable CTCs suitable for downstream analysis. In summary, these advancements underscore the importance of developing multifaceted approaches that address the inherent heterogeneity of CTCs. By leveraging a combination of molecular and physical characteristics, future liquid biopsy platforms can achieve higher sensitivity and specificity, ultimately enhancing their clinical utility in personalized cancer management.

## Figures and Tables

**Table 1:** Utilization of CTCs in clinical practice.

Early cancer detection [36]	Detecting CTCs in blood samples can facilitate early diagnosis of cancers, potentially before symptoms arise. This non-invasive approach allows for timely intervention, which is crucial for improving patient outcomes.
Prognosis prediction [18]	The number and characteristics of CTCs correlate with disease stage and aggressiveness. Elevated CTC counts are often associated with a higher risk of metastasis and poorer prognosis. Monitoring CTC levels can help to predict disease progression and informed treatment decisions.
Monitoring treatment response [37]	CTC analysis enables real-time assessment of tumor response to therapy. A decrease in CTC count during treatment may indicate a positive response, while an increase could suggest resistance or disease progression. This dynamic monitoring can aid in adjusting treatment plans promptly.
Detecting minimal residual disease [38]	After surgical removal of tumors, CTCs can persist in the bloodstream, leading to recurrence. Identifying these residual cells through CTC analysis allows for early intervention to prevent relapse.
Assessing metastatic potential [39]	CTCs are essential for understanding the metastatic process. Their presence and characteristics can provide insights into the likelihood of cancer spreading to other parts of the body, guiding surveillance and preventive strategies.
Personalized medicine [40]	Analyzing CTCs allows for molecular profiling of tumors, identifying specific mutations and alterations. This information is crucial for selecting targeted therapies tailored to the individual patient’s cancer, enhancing treatment efficacy.

**Table 2:** Summary of discussed CTC enrichment technologies.

EpCAM-dependent CTC enrichment	Positive selection	CELLSEARCH^®^ [59]	The first FDA-approved system for isolating and enumerating circulating tumor cells (CTCs) in patients with metastatic breast, prostate, or colorectal cancer.
		IsoFlux™ CTC System [61]	A microfluidic-based system that captures CTCs using immunomagnetic beads targeting the EpCAM, enabling high-purity isolation for downstream analysis.
		GILUPI CellCollector^®^ [65]	An anti-EpCAM coated medical-grade catheter, which is inserted into a vein, allowing for in vivo isolation.
		GenoCTC [111]	The device utilizes microfluidic magnetophoresis and a specialized isolation chip with optimized ferromagnetic wire patterns to enrich CTCs, targeting both epithelial and mesenchymal markers.
EpCAM-independent CTC enrichment	Positive selection	On-chip Sort [114]	The device utilizes fluorescence-based cell sorting for the positive selection of different cell types in the samples.
	Negative selection	Dynabeads™ [131]	Magnetic beads coated with antibodies against CD45, enabling the depletion of leukocytes from blood samples to enrich CTCs.
		RosetteSep™ [132]	A negative selection method that uses tetrameric antibody complexes to remove unwanted blood cells, facilitating the isolation of CTCs.
		CTC-iCHIP [127]	A microfluidic device that combines inertial focusing and magnetic separation to isolate CTCs from whole blood without the need for labeling.
	Label-free enrichment	Parsortix^®^ [147]	A microfluidic system that separates CTCs from blood based on size and deformability, allowing for label-free isolation.
		ClearCell^®^ FX1 System [152]	A microfluidic device that captures CTCs using size-based filtration, enabling label-free isolation for subsequent analysis.

## Data Availability

Not applicable.
